# A field guide to cultivating computational biology

**DOI:** 10.1371/journal.pbio.3001419

**Published:** 2021-10-07

**Authors:** Gregory P. Way, Casey S. Greene, Piero Carninci, Benilton S. Carvalho, Michiel de Hoon, Stacey D. Finley, Sara J. C. Gosline, Kim-Anh Lȇ Cao, Jerry S. H. Lee, Luigi Marchionni, Nicolas Robine, Suzanne S. Sindi, Fabian J. Theis, Jean Y. H. Yang, Anne E. Carpenter, Elana J. Fertig

**Affiliations:** 1 Imaging Platform, Broad Institute of MIT and Harvard, Cambridge, Massachusetts, United States of America; 2 Center for Health AI, University of Colorado School of Medicine, Aurora, Colorado, United States of America; 3 RIKEN Center for Integrative Medical Sciences Yokohama, Kanagawa, Japan; 4 Human Technopole, Milan, Italy; 5 Department of Statistics, Institute of Mathematics, Statistics and Scientific Computing, University of Campinas, Campinas, Brazil; 6 Department of Biomedical Engineering, Quantitative and Computational Biology, and Chemical Engineering & Materials Science, University of Southern California, Los Angeles, California, United States of America; 7 Pacific Northwest National Laboratory, Seattle, Washington, United States of America; 8 Melbourne Integrative Genomics, School of Mathematics and Statistics, The University of Melbourne, Melbourne, Australia; 9 Ellison Institute and Departments of Medicine/Oncology, Chemical Engineering, and Material Sciences, University of Southern California, Los Angeles, California, United States of America; 10 Department of Pathology and Laboratory Medicine, Weill-Cornell Medicine, New York, New York, United States of America; 11 Computational Biology Lab, New York Genome Center, New York, New York, United States of America; 12 Department of Applied Mathematics, University of California Merced, Merced, California, United States of America; 13 Institute of Computational Biology, Helmholtz Center Munich and Department of Mathematics, Technical University of Munich, Munich, Germany; 14 Charles Perkins Centre and School of Mathematics and Statistics, The University of Sydney, Australia; 15 Convergence Institute, Departments of Oncology, Biomedical Engineering, and Applied Mathematics and Statistics, Johns Hopkins University, Baltimore, Maryland, United States of America

## Abstract

Evolving in sync with the computation revolution over the past 30 years, computational biology has emerged as a mature scientific field. While the field has made major contributions toward improving scientific knowledge and human health, individual computational biology practitioners at various institutions often languish in career development. As optimistic biologists passionate about the future of our field, we propose solutions for both eager and reluctant individual scientists, institutions, publishers, funding agencies, and educators to fully embrace computational biology. We believe that in order to pave the way for the next generation of discoveries, we need to improve recognition for computational biologists and better align pathways of career success with pathways of scientific progress. With 10 outlined steps, we call on all adjacent fields to move away from the traditional individual, single-discipline investigator research model and embrace multidisciplinary, data-driven, team science.

## Abstract 2

Do you want to attract computational biologists to your project or to your department? Despite the major contributions of computational biology, those attempting to bridge the interdisciplinary gap often languish in career advancement, publication, and grant review. Here, sixteen computational biologists around the globe present "A field guide to cultivating computational biology," focusing on solutions.

Biology in the digital era requires computation and collaboration. A modern research project may include multiple model systems, use multiple assay technologies, collect varying data types, and require complex computational strategies, which together make effective design and execution difficult or impossible for any individual scientist. While some labs, institutions, funding bodies, publishers, and other educators have already embraced a team science model in computational biology and thrived [1–7], others who have not yet fully adopted it risk severely lagging behind the cutting edge. We propose a general solution: “deep integration” between biology and the computational sciences. Many different collaborative models can yield deep integration, and different problems require different approaches (**Fig 1**).

**Fig 1:**
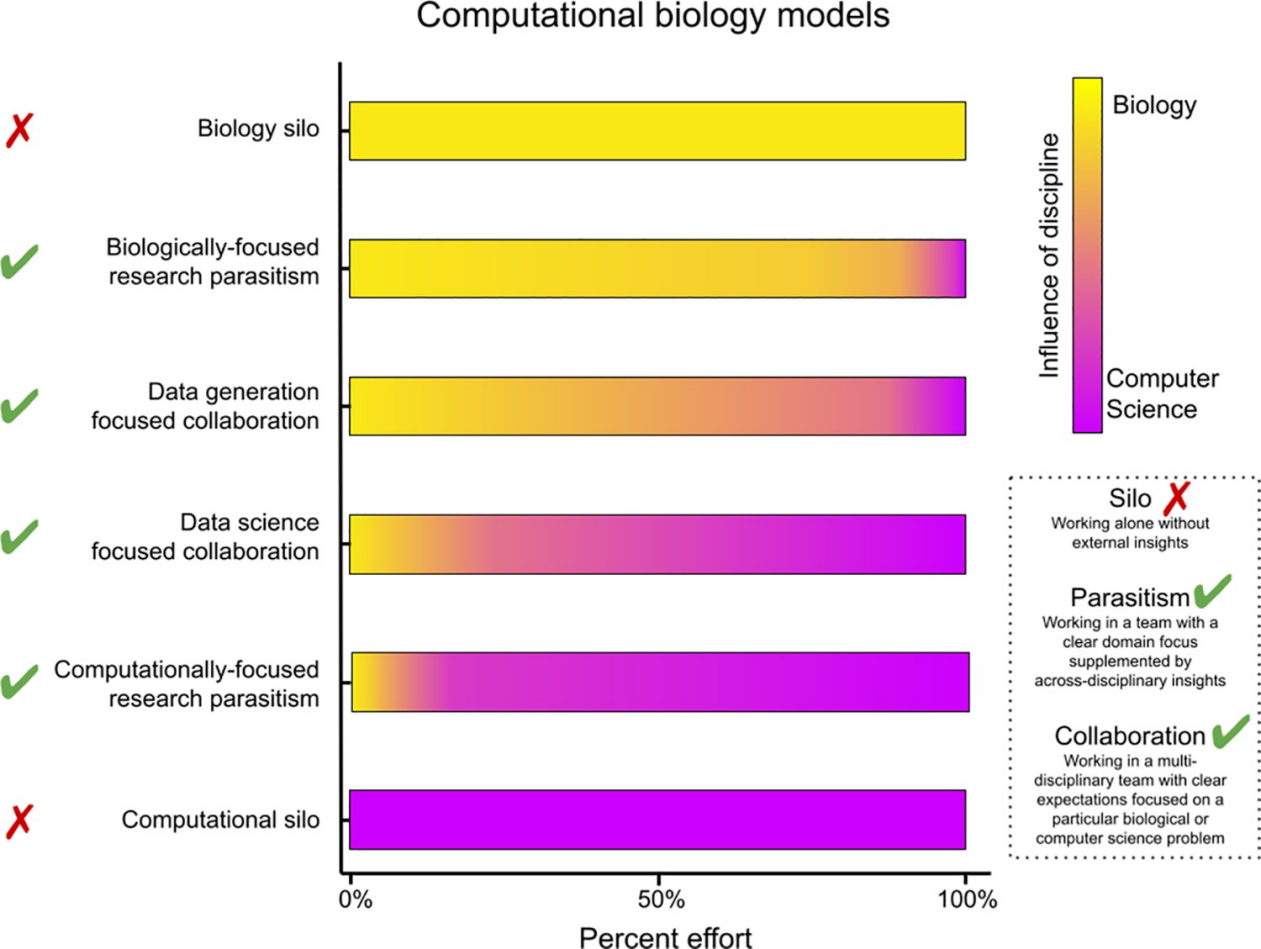
Supporting interdisciplinary team science will accelerate biological discoveries. Scientists who have little exposure to different fields build silos, in which they perform science without external input. To solve hard problems and to extend your impact, collaborate with diverse scientists, communicate effectively, recognize the importance of core facilities, and embrace research parasitism. In biologically focused parasitism, wet lab biologists use existing computational tools to solve problems; in computationally focused parasitism, primarily dry lab biologists analyze publicly available data. Both strategies maximize the use and societal benefit of scientific data.

In this article, we define computational science extremely broadly to include all quantitative approaches such as computer science, statistics, machine learning, and mathematics. We also define biology broadly, including any scientific inquiry pertaining to life and its many complications. A harmonious deep integration between biology and computer science requires action—we outline 10 immediate calls to action in this article and aim our speech directly at individual scientists, institutions, funding agencies, and publishers in an attempt to shift perspectives and enable action toward accepting and embracing computational biology as a mature, necessary, and inevitable discipline (**Box 1**).

**Box 1. Ten calls to action for individual scientists, funding bodies, publishers, and institutions to cultivate computational biology. Many actions require increased funding support, while others require a perspective shift. For those actions that require funding, we believe convincing the community of need is the first step toward agencies and systems allocating sufficient support**

1. Respect collaborators’ specific research interests and motivations
  Problem: Researchers face conflicts when their goals do not align with collaborators. For example, projects with routine analyses provide little benefit for computational biologists.
  Solution: Explicit discussion about interests/expertise/goals at project onset.
  Opportunity: Clearly defined expectations identify gaps, provide commitment to mutual benefit.
2. Seek necessary input during project design and throughout the project life cycle
  Problem: Modern research projects require multiple experts spanning the project’s complexity.
  Solution: Engage complementary scientists with necessary expertise throughout the entire project life cycle.
  Opportunity: Better designed and controlled studies with higher likelihood for success.
3. Provide and preserve budgets for computational biologists’ work
  Problem: The perception that analysis is “free” leads to collaborator budget cuts.
  Solution: When budget cuts are necessary, ensure that they are spread evenly.
  Opportunity: More accurate, reproducible, and trustworthy computational analyses.
4. Downplay publication author order as an evaluation metric for computational biologists
  Problem: Computational biologist roles on publications are poorly understood and undervalued.
  Solution: Journals provide more equitable opportunities, funding bodies and institutions improve understanding of the importance of team science, scientists educate each other.
  Opportunity: Engage more computational biologist collaborators, provide opportunities for more high-impact work.
5. Value software as an academic product
  Problem: Software is relatively undervalued and can end up poorly maintained and supported, wasting the time put into its creation.
  Solution: Scientists cite software, and funding bodies provide more software funding opportunities.
  Opportunity: More high-quality maintainable biology software will save time, reduce reimplementation, and increase analysis reproducibility.
6. Establish academic structures and review panels that specifically reward team science
  Problem: Current mechanisms do not consistently reward multidisciplinary work.
  Solution: Separate evaluation structures to better align peer review to reward indicators of team science.
  Opportunity: More collaboration to attack complex multidisciplinary problems.
7. Develop and reward cross-disciplinary training and mentoring
  Problem: Academic labs and institutions are often insufficiently equipped to provide training to tackle the next generation of biological problems, which require computational skills.
  Solution: Create better training programs aligned to necessary on-the-job skills with an emphasis on communication, encourage wet/dry co-mentorship, and engage younger students to pursue computational biology.
  Opportunity: Interdisciplinary students uncover important insights in their own data.
8. Support computing and experimental infrastructure to empower computational biologists
  Problem: Individual computational labs often fund suboptimal cluster computing systems and lack access to data generation facilities.
  Solution: Institutions can support centralized compute and engage core facilities to provide data services.
  Opportunity: Time and cost savings for often overlooked administrative tasks.
9. Provide incentives and mechanisms to share open data to empower discovery through reanalysis
  Problem: Data are often siloed and have untapped potential.
  Solution: Provide institutional data storage with standardized identifiers and provide separate funding mechanisms and publishing venues for data reuse.
  Opportunity: Foster new breed of researchers, “research parasites,” who will integrate multimodal data and enhance mechanistic insights.
10. Consider infrastructural, ethical, and cultural barriers to clinical data access
  Problem: Identifiable health data, which include sensitive information that must be kept hidden, are distributed and disorganized, and thus underutilized.
  Solution: Leadership must enforce policies to share deidentifiable data with interoperable metadata identifiers.
  Opportunity: Derive new insights from multimodal data integration and build datasets with increased power to make biological discoveries.

## Respect collaborators’ specific research interests and motivations

Computational biology hinges on mutual respect between scientists from different disciplines, and key elements of respect are understanding a colleague’s particular expertise and motivation. Individual scientists cannot stick strictly to their “home” discipline or treat one as working in service of another. Computationalists do not like to be seen as “just” running the numbers any more than biologists appreciate the perception that they are “just” a pair of hands that produced the data. Statistics, database structures, clinical informatics, genetics, epigenetics, genomics, proteomics, imaging, single-cell technologies, structure prediction, algorithm development, machine learning, and mechanistic modeling are all distinct fields. Biologists should not be offended if a particular idea does not fit a computational biologist’s research agenda, and computational scientists need to clearly communicate analysis considerations, approaches, and limitations.

Some institutions subsidize core facilities, which offer a variety of data collection and analytical services across a spectrum of data types. While some core services can carry out custom analyses and collect novel data types, others may be limited to standardized analysis and data collection pipelines due to their mission, bandwidth, or expertise. US National Laboratories are an unusual environment; unlike most core facilities, scientific careers are focused on technology development and benefit from internally allocated funding for their own research programs. As a community, we must value critical data and insights contributed by core facility staff by including them as authors.

Certain grant mechanisms can provide flexibility for computational biologists to develop new technologies, but the scope often focuses on method development, limiting the ability to collaborate on application-oriented projects. The current academic systems incentivize mechanism and translational discovery for biology but methodological or theoretical advances for computational sciences. This explains a common disconnect when collaborating: Projects that require routine use of existing methodology typically provide little benefit to the computational person’s academic record no matter how unique a particular dataset.

Therefore, we urge team science practitioners to conduct a transparent and explicit discussion of each investigator’s expertise, limitations, goals, expectations, deliverables, and publication strategies upfront. Matching research interests can facilitate dual submission of methodological and biological manuscripts, which provides leading roles for all investigators in the research team.

## Seek necessary input during project design and throughout the project life cycle

Interdisciplinary projects without sufficient planning risk wasting time and resources. Scientists lacking particular expertise for a project should engage collaborators with such expertise from the beginning of the research project lifecycle [8]. Computational scientists may have critical insights that impact the scope of the biological questions, study design, analysis, and interpretation. Similarly, biologists’ early involvement may influence the algorithmic approach, data visualization, and refinement of analysis. The onset of a project is the ideal time to plot out feasibility, brainstorm solutions, and avoid costly missteps. It is also the time to establish clear communication and define expectations and responsibilities, in particular in the gray area between (experimental) data generation and (computational) data analysis.

Computational scientists should learn about data acquisition techniques and the factors that influence data quality, as well as the cost to collect new datasets. As the project progresses, collaborators must understand that data analysis is rarely turnkey: Optimal analysis requires iteration and engagement and can yield fruitful discovery and new questions to ask.

## Provide and preserve budgets for computational biologists’ work

There is a common misconception that the lack of physical experimentation and laboratory supplies makes computational work automated, quick, and inexpensive. This stems from a sense that labor/time is a “free” resource in academia, and perhaps that each analysis should only need to be run a single time. In reality, even for well-established data types, analysis can often take as much or more time and effort to perform as generating the data at the bench. Moreover, it typically also requires pipeline optimization, software development and maintenance, and user interfaces so that methods remain usable beyond the scope of a single publication or project. Given that computational biologists often command higher salaries due to competition with industry, researchers should ensure space in the budget to support these efforts and computing costs for projects.

Scientists, institutions, and funders should also preserve budgets for collaborative researchers. When funding agencies impose budget cuts, computational collaborators’ budgets are often the first to go. This can substantially impact computational laboratories’ ability to provide independent projects and salaries to their trainees and staff members, especially considering that time spent providing preliminary analyses or ideas for the proposal cannot be recouped. In one computational biology laboratory, one-third of its collaborators’ funded grants cut their budget entirely, and another third cut their budget partially—by an average of 90% [9]. Although principal investigators are authorized to make budget changes at will, they should consider the impact of doing so on the long-term health of their relationship with their computational collaborators and the scientific community more generally. Institutions and agencies can help promote good behavior by a simple policy change: By default, budget cuts are distributed evenly; the lead investigator can then propose changes if needed. Societies should also advocate for this change at the funding agencies.

## Downplay publication author order as an evaluation metric for computational biologists

**Fig 2:**
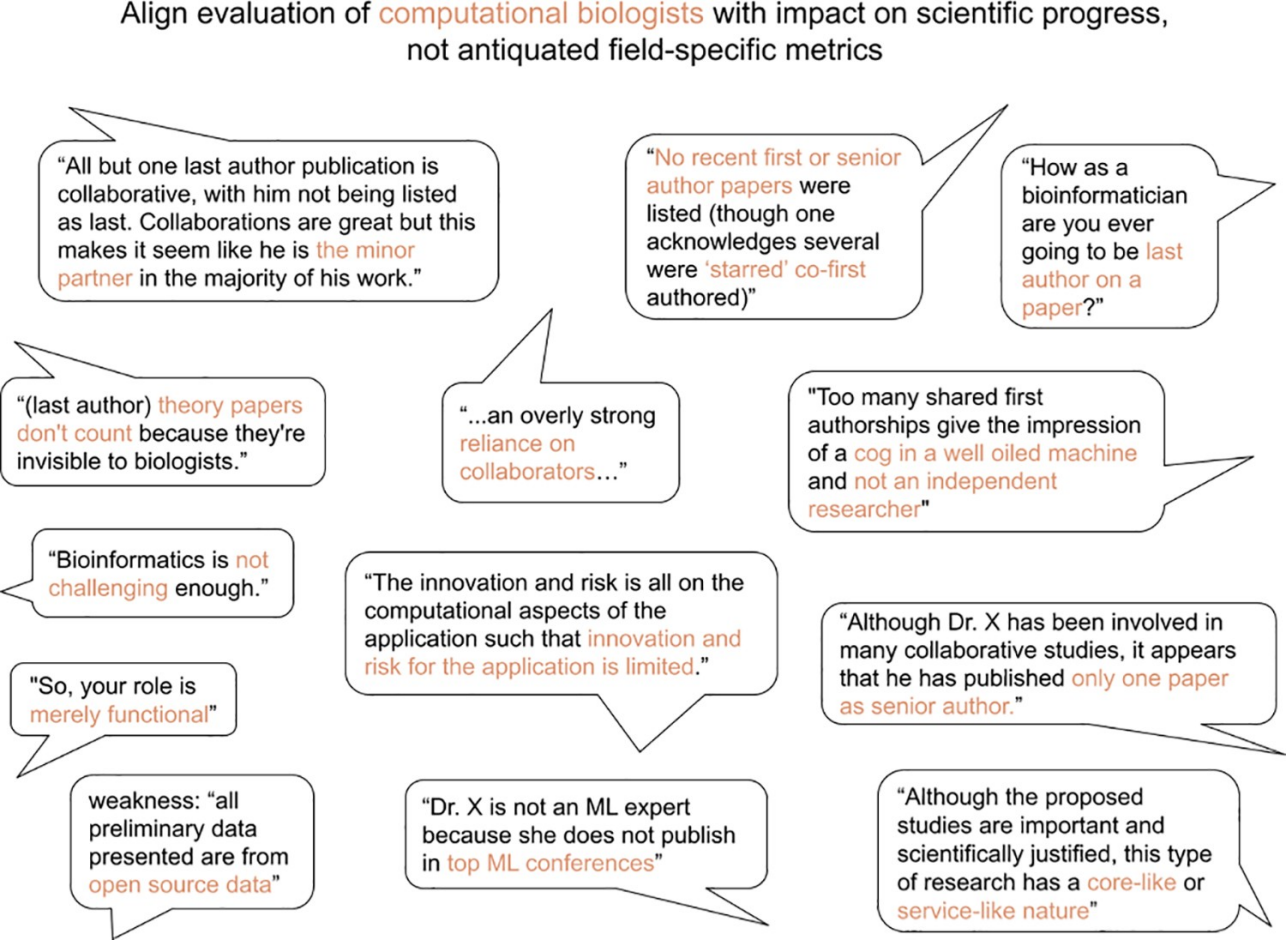
Reviewers, institutions, and funding agencies often evaluate computational biologists ineffectively. Everyone receives bad reviews; most are unavoidable. However, we notice a consistent trend with reviewers from disparate fields not being equipped to understand, evaluate, or appreciate the importance of computational biology contributions to team science. We collected these critiques via Twitter and received permission to directly quote.

Many high-impact papers have computational biologists in key authorship positions. In biology journals, these are customarily the first and last positions. Middle-author placements are very common for computational biologists, reflecting a methodological contribution on a paper to address a particular biological question. Co-first or co-senior authorships provide a means to provide credit when computational contributions are equally important to a paper, but such designations are often dramatically discounted in grant, hiring, promotion, and tenure assessments. For example, we have seen recent comments such as: “No recent first or senior author papers were listed (though one acknowledges several were ‘starred’ co-first authored)” (**Fig 2**).

Although the idea of breaking away from a linear, rank-ordered list of authors may seem unimaginable, we note that the biology journal standards of author order are not universal. In fields such as mathematics and physics, authors are listed alphabetically. These differences further complicate evaluation of computational publication records by biologists, who are typically not aware of the relative reputation of computational publishing venues (and vice versa). Other author ordering alternatives are possible, and scientific publishing has experienced dramatic change in recent years [10]. One option is for journals to formally encourage swapping the order of authors that have been designated as “equally contributing” via their display interfaces. Another might be to allow designations such as the “corresponding author on experimental aspects” and the “corresponding author on bioinformatics.”

Fundamentally, these issues will only be solved through educating institutional leaders and grant/paper reviewers on how to best understand and appreciate different kinds of scientists’ contributions. From calling computational biologists “research parasites” [11] to “mathematical service units” [2], it is clear that we have a long way to go (**Fig 2**). Institutions can address this by altering promotion structures to depend upon an author’s contributions, regardless of order in the publication. One innovative solution was undertaken in 2021 by the Australian Research Council (ARC): A 500-word section was added to proposals for applicants to describe “research context” and “explain the relative importance of different research outputs and the expectations” in the specific discipline/s of the applican10 ten most relevant scientific results (articles, registered software, patents, and other items that can be chosen at the author’s discretion) and verifiable description of the impact of these items (citations, prizes, impact in public policies, and so on). The Royal Society Resume for Researchers was also designed to support and highlight a variety of research contributions [12]. This provides an opportunity to explain author order and other information helpful to assess merit within a discipline.

## Value software as an academic product

Another complication to evaluating computational biologists is that their primary research output may not be papers but instead valuable software or data [13]. The US National Institutes of Health changed its biosketch format to explicitly encourage software products to be mentioned in its “Contributions to Science” section, where usage and impact can be described. It is becoming more common to publish software and data papers, aided in part by some journals creating article types specifically for software and data. Allowing updated versions of software to also be published provides academic credit for the largely thankless task of software maintenance over time.

Schemes for tracking the usage of software and data are a work in progress [14]. Citation counts for papers are a key metric used in career decisions but are inconsistent for software and data papers. Every scientist can help ensure that software and data creators receive credit for their work by citing the related papers rather than just mentioning the name of the software, website, or database (or omitting mention entirely), which leads to inconsistent tracking when evaluating investigator impact [15]. Journals can require that software and data are properly cited.

Quality software development and maintenance is crucial for efficient and reproducible data analysis and is a key ingredient for successful computational biology projects [16]. Community-level software ecosystems and pipeline-building tools have outsized impact because they standardize analyses and minimize software development costs for individual labs [13]. Yet, academic systems, which prioritize innovation, commonly undervalue software maintenance and development. Software products funded across projects, such as slurm or singularity at US National Laboratories, have provided valuable resources for the broader scientific community [17,18]. These projects were initially funded as independent tools, which supported independent computational biology labs. Software has a major impact on the progress of science but is underfunded by many agencies. The few agencies that do fund software maintenance are spread thin, given the global demand [19].

## Establish academic structures and review panels that specifically reward team science

Wet lab biologists trained in traditional evaluation schemes can be quick to dismiss a researcher with a lack of a single driving biological question for the laboratory, many middle-authored papers, publication in computational conferences rather than journals, a low citation count or h-index (due to field-specific differences), or funding through grants led by others, leading to comments such as “How do people like you ever get last-author papers?” and “an overly strong reliance on collaborators” [2]. Computational scientists can dismiss a body of work as too applied, with not enough theory or conference papers that are the currency of the field. Evaluation panels should therefore include interdisciplinary researchers and be provided with guidelines about the challenges of interdisciplinary research. If, for example, middle authorships are seen by review committees as worthless (even, in some reported cases, detrimental) to a publication record, major contributors to the progress of science will go “extinct,” unable to get their research funded. It is therefore important for institutions to learn to appreciate the value of many small contributions versus a few large contributions.

We hope to reach a day where quantitative skills are so pervasive (and valued) that calling someone a “computational biologist” sounds just as odd as a “pipette biologist” [2]. In the meantime, establishing separate structures and review schemes is another approach to support this class of researchers. To promote faculty success, many institutions have created Systems Biology, Computational Biology, or Biomedical Informatics departments to provide an environment in which researchers can thrive and be evaluated by like-minded interdisciplinary colleagues. Similarly, interdisciplinary journals, grant review panels, and funding schemes support the publication and funding of work evaluated by peers. Some organizations are already promoting team science efforts by shifting cultures in recognition, funding, and career development, such as the UK Academy of Medical Sciences and National Research Council [20].

Likewise, institutions should take care to ensure that interdisciplinary researchers are recognized and rewarded for contributions across disciplines and departments, for example, through the evaluation system, additional compensation, and supplemental administrative staff. These researchers face more than their fair share of demands from collaborative roles on grants, administrative leadership, educational initiatives, thesis committees, and consultations. Many are jointly appointed to 2 or more departments, introducing additional service requirements that are invisible to each individual department [21]. Computational biologists can struggle to prioritize their research and these demands—even more so if they are in an underrepresented demographic, as such scientists face disproportionate demands on their time and disproportionate costs (being labeled uncollaborative and unsupportive) if they decline. Instead of ignoring or even negatively assessing team science contributions, promotion and tenure committees should include criteria that are indicators of success in collaborative-style work, including effort level as co-investigator on grants, core facility leadership, collaborative authorship contributions, and community service.

## Develop and reward cross-disciplinary training and mentoring

As large datasets become increasingly common, computational expertise is a necessary asset for any biologist. The deepest insights often result from data analyzed by the biologists who designed and conducted the experiment. Likewise, an understanding of biological data is a necessary asset for any computer scientist working on biological data. The most impactful methodological leaps often result from a computer scientist with a deep understanding of the nuances and limitations of particular data. Institutions can help hybrid trainees bridge gaps through computational biology training, strategic organization of physical space, and team science–oriented evaluation metrics for mentors.

The first step in acquiring practical computational biology skills is to become comfortable with the basics in the unfamiliar domain: either programming or biology. Societies and other nonprofits can play a major role here; examples include the iBiology, Software Carpentries, CABANA in Latin America, and NEUBIAS in Europe [22–25]. Still, institutions must also explicitly support cross-disciplinary training and mentoring. Institutions can provide educational opportunities focused on basic programming, data analysis, and reproducibility, as well as core biology principles chosen by unique institutional strengths. Teaching collaborative and interdisciplinary skills ideally begins at the undergraduate level where courses should be redesigned to blend computer science and biology.

**Fig 3:**
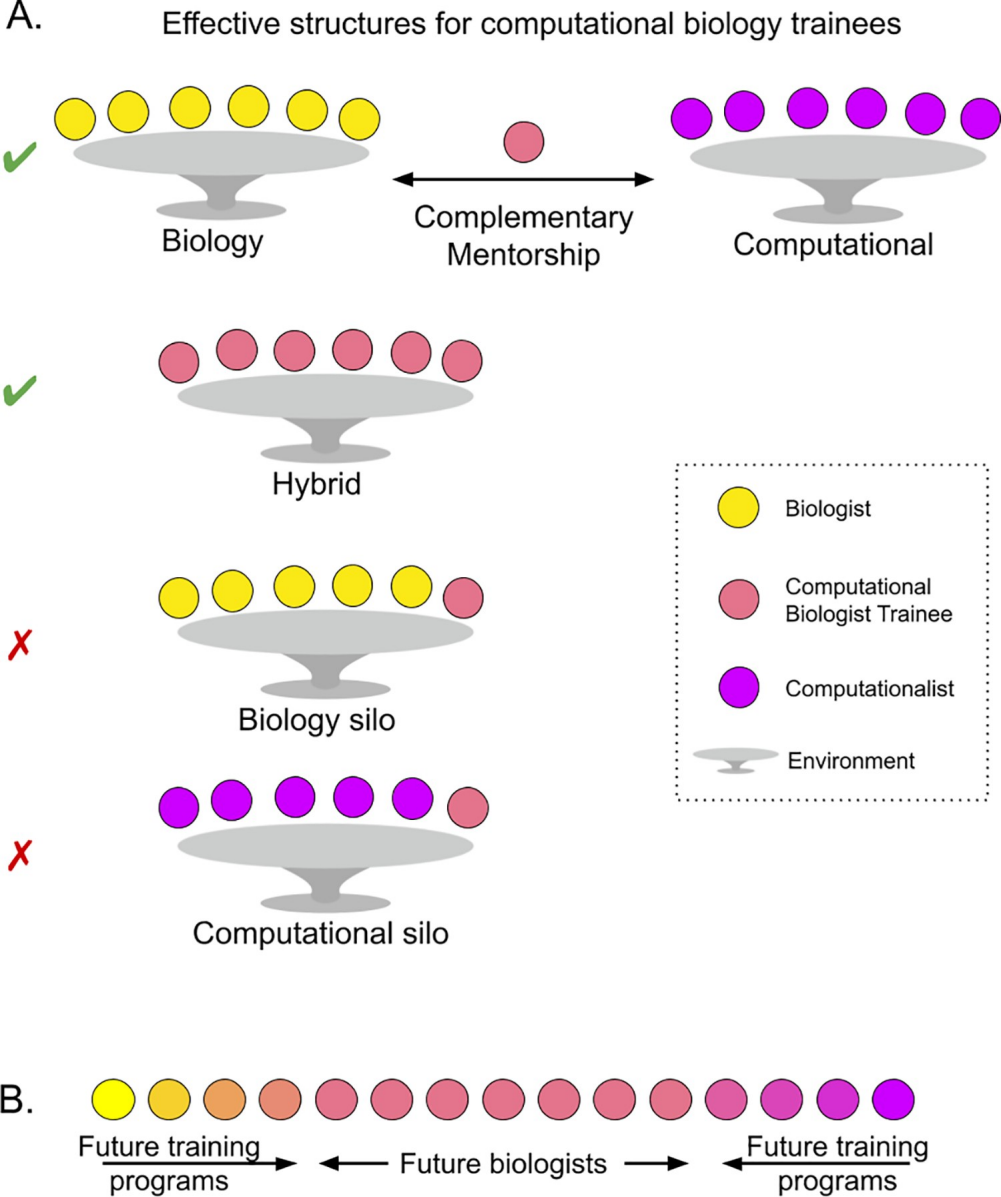
Promoting healthy computational biology environments and training programs will empower future biologists. **(A)** Existing biology and computational labs can cooperate to provide complementary mentorship to computational biologists, and hybrid labs can provide sufficient support. Institutions must provide oversight to ensure that ill-equipped labs have trainees’ career goals in mind and do not view them as inexpensive labor. **(B)** Computational biology programs are at an advantage to provide necessary training to forge the biologists of the future.

Institutions can also organize spaces to facilitate deep integration between biology and computer science. Combining wet and dry lab spaces encourages interactions among researchers with diverse expertise. For computational biology trainees who are embedded in laboratories with a focused and single-discipline research agenda, institutions and lab heads can seek mentors from complementary domains, and these external mentors should be rewarded institutionally. We urge institutional oversight for trainees hired by individual out-of-discipline PIs to be sure that they are learning in their chosen field and not treated as inexpensive hired hands. We also emphasize that not all labs require supplemental trainee supervision and mentorship; many lead investigators in computational biology are quite experienced enough to cover both sides (**Fig 3**).

A challenge of being interdisciplinary in many current academic structures is facing evaluation according to often conflicting, field-specific evaluation metrics that often fail to incentivize meaningful contributions to scientific progress. Mentors and departments should work to change evaluation systems to reward team science and, in the meanwhile, guide computational biology trainees to ensure that their career goals can be met. Furthermore, mentors and departments should emphasize communication skills, through explicit focus on cross-disciplinary conversations, brainstorming, and presentations. For example, training programs at National Labs typically require that trainees meet regularly with those outside of their primary area. Within academic departments, offices exclusively focused on scientific communication empower scientists to reach out across disciplines and to the public.

## Support computing and experimental infrastructure to empower computational biologists

Computing infrastructure (whether local cluster, volunteer, or cloud-based) is essential to modern biomedical data science and requires compute power, data storage, networking, and system administration—all of which can introduce significant costs to research projects. For most institutions, this computing infrastructure is often not centrally provided nor well supported; it often falls through the cracks between institutional information technology (IT) and research offices. As a result, individual laboratories often independently (and inefficiently) fund their own infrastructure and perform systems administration for clusters or the cloud that are far outside their actual expertise. Institutions that wish to foster computational research should subsidize infrastructure costs and provide support staff to assist in its use. Some funding agencies already offer grant mechanisms that support computing hardware or cloud-based computing, such as the US STRIDES initiative, but others should also recognize these necessary costs and increase budgets to better support computational biology.

Historically, computational biologists relied on collaborators or public resources for datasets. It is now becoming more common for researchers with an informatics background to be running experiments, whether as a primary data source or to benchmark new technologies, validate algorithms, and test predictions and theories. Being able to bridge the dry lab–wet lab gap can have a major impact on a computational biologist’s success. Institutions and laboratory neighbors can flexibly offer laboratory space for computational biologists when needed, and they can offer appropriate biology mentors for computational biologists joining computational groups. Core facilities can extend support: for example, rather than only ingesting fully prepped samples or only providing instrument time, facilities can offer full service (from sample prep to data acquisition) to computational laboratories for an additional fee.

## Provide incentives and mechanisms to share open data to empower discovery through reanalysis

“Data available upon request” is outdated, ineffective [26], and should be outlawed in publications (privacy reasons excepted, see next section). Data and code often become more valuable over time with new techniques or complementary new data. Indeed, some of the most challenging problems facing biological and clinical research are only possible through access to well-curated, large-scale datasets. Funding agencies, publishers, and the scientific community must continue to recognize both dissemination and reanalysis of reusable data as an impactful research output. Findable, accessible, interoperable, and reusable (FAIR) principles have already been adopted and even required by many funding agencies and organizations [27]. New “Resource” article types have been introduced in many journals, and entirely new journals (e.g., Gigascience, Scientific Data, and Data in Brief) launched to provide a publication route, yielding academic credit for the creation, organization, and sharing of useful datasets.

Explicit funding mechanisms aimed at data reuse can also facilitate algorithm development. These mechanisms can range from grant applications specifically targeting dataset reuse, a hackathon hosted by a disease foundation, or even a DREAM challenge in which the organizers curate a dataset specifically for algorithm development. These exercises also encourage the next generation of researchers to challenge, or augment, conclusions reported in original marker papers. Institutions can also fund internal algorithm development efforts to improve analyses of commonly generated data.

Data-generating biologists can share data types through a growing ecosystem of repositories [28]. Logistically, storing and disseminating data is a complex task, even for those with computational skills; it requires effective communication and knowledge encompassing IT, database structures, security/privacy, and desktop support that is distinct from analysis and the development of computational methods. Institutions and funding agencies should provide specialists in this and develop interfaces with consistent ontologies to ease the process and facilitate data reuse. Creating mechanisms for capturing metadata and incentives that support high-quality annotations can help to return more value from computational analyses [29,30]. To maximize the engagement of computational biologists, it is often helpful to provide data in a raw form as well as commonly used summary forms—controlling access as required to meet ethical and legal constraints [28,31].

## Consider infrastructural, ethical, and cultural barriers to clinical data access

Institutions with large medical centers are realizing the promise of discovery from multimodal datasets of their unique patient cohorts, including primary clinical data as well as data from corresponding biospecimens using emerging molecular and imaging technologies. For example, deep learning has the potential to revolutionize pathology, but sufficient data are needed, often thousands of annotated images spanning patient groups. Infrastructural, ethical, and cultural considerations all place barriers to large-scale data access for computational research.

Institutions can play a role in supporting data access by providing centralized database structures that automatically ingest patient electronic health records and research-level data collected on these patients [32]. This requires careful attention to privacy, such that access to data is provided only to legally authorized researchers. It also requires policies in place governing whether patients will be notified about any potential health risks uncovered based on their data. Ethicists and clinical societies play a critical role in developing appropriate guidelines for such computational research on patient datasets that consider patient privacy and potential for improved public health. Inclusion of demographic information in these datasets and outreach to underrepresented populations is critical to overcome biases in data-driven discovery and introduces further ethical considerations.

The current system rewards scientists and institutions that closely guard clinical datasets, because exclusive analysis can benefit careers and the data can be monetized due to commercial demand for novel biomarkers and therapeutic targets. Strong leadership is essential to incentivize academic investigators to collect datasets in a coordinated way across disease groups to enable research for public benefit. Federated learning, where machine learning models can be trained on multiple datasets without actually sharing the raw data, may work around some barriers.

## Conclusions

Visionaries a decade ago aspired to bridge the domains of computational sciences and biology [1–7]. Since then, computational biology has emerged as a mature scientific discipline. It’s time that traditional academic schemes built for the era of single-discipline biology evolve to support the interdisciplinary team science necessary for human progress.

As computational biology has grown rapidly over the last 30 years, we may ask what computational biology will look like 30 years from now, if cultures shift in the ways we propose.

We foresee the ever-growing amount of data and associated analytical questions outstripping the supply of researchers with computational skills. This unmet demand will drive wet lab biologists to use software, which funding bodies and publishers should support to become more optimized, reproducible, maintainable, and easy to use without relying on a dedicated computationalist. Likewise, as more data become open and interoperable and as contracted wet lab facilities grow, computationalists may not need to rely on dedicated wet lab scientists.

In the interim, as the field continues to develop new methods and biological data types, we foresee research parasitism and team science collaborations flourishing, and the scientists and institutions who focus most on cultivating computational biology will be rewarded.

Over time, the distinction between wet and dry biologists may fade, as both are working toward a common goal of understanding biology, and hybrid biologists [2,33] will emerge who understand the importance of collaboration and are equally adept at the experimental and the computational aspects of biology.

## References

[1] Loman N, Watson M. So you want to be a computational biologist? Nat Biotechnol. 2013;31:996–8. doi: 10.1038/nbt.2740 24213777

[2] Markowetz F. All biology is computational biology. PLoS Biol. 2017;15:e2002050. doi: 10.1371/journal.pbio.2002050 28278152 PMC5344307

[3] Form D, Lewitter F. Ten simple rules for teaching bioinformatics at the high school level. PLoS Comput Biol. 2011;7:e1002243. doi: 10.1371/journal.pcbi.1002243 22046117 PMC3203051

[4] Ayoob JC, Kangas JD. 10 simple rules for teaching wet-lab experimentation to computational biology students, i.e., turning computer mice into lab rats. PLoS Comput Biol. 2020;16:e1007911. doi: 10.1371/journal.pcbi.1007911 32497035 PMC7271982

[5] Demharter S, Pearce N, Beattie K, Frost I, Leem J, Martin A, et al. Ten simple rules for surviving an interdisciplinary PhD. PLoS Comput Biol. 2017;13:e1005512. doi: 10.1371/journal.pcbi.1005512 28542231 PMC5444598

[6] Mulder N, Schwartz R, Brazas MD, Brooksbank C, Gaeta B, Morgan SL, et al. The development and application of bioinformatics core competencies to improve bioinformatics training and education. PLoS Comput Biol. 2018;14:e1005772. doi: 10.1371/journal.pcbi.1005772 29390004 PMC5794068

[7] Carey MA, Papin JA. Ten simple rules for biologists learning to program. PLoS Comput Biol. 2018;14:e1005871. doi: 10.1371/journal.pcbi.1005871 29300745 PMC5754048

[8] Integrating Quantitative Approaches in Cancer Research and Oncology. Trends Cancer Res. 2021;7:270–5. doi: 10.1016/j.trecan.2021.01.011 33637445

[9] Carpenter A. Scientific collaborators are not disposable. 2018 Aug 9 [cited 2020 Dec 7]. Available from: https://www.timeshighereducation.com/opinion/scientific-collaborators-are-not-disposable

[10] Callaway E. Will the pandemic permanently alter scientific publishing? Nature. 2020;582:167–8. doi: 10.1038/d41586-020-01520-4 32504015

[11] Longo DL, Drazen JM. Data Sharing. N Engl J Med. 2016:276–7. doi: 10.1056/NEJMe1516564 26789876

[12] Research culture: Résumé for Researchers. [cited 2021 Jul 23]. Available from: https://royalsociety.org/blog/2019/10/research-culture/

[13] Perkel JM. Ten computer codes that transformed science. Nature. 2021;589:344–8. doi: 10.1038/d41586-021-00075-2 33473232

[14] Wade AD, Williams I. CORD-19 Software Mentions. Dryad. 2021. doi: 10.5061/dryad.vmcvdncs0

[15] Singh CD. The unsung heroes of scientific software. Nature. 2016;529:115–6. doi: 10.1038/529115a 26738597

[16] Levet F, Carpenter AE, Eliceiri KW, Kreshuk A, Bankhead P, Haase R. Developing open-source software for bioimage analysis: opportunities and challenges. F1000Res. 2021;10:302. doi: 10.12688/f1000research.52531.1 34249339 PMC8226416

[17] Kurtzer GM, Sochat V, Bauer MW. Singularity: Scientific containers for mobility of compute. PLoS ONE. 2017;12:e0177459. doi: 10.1371/journal.pone.0177459 28494014 PMC5426675

[18] Yoo AB, Jette MA, Grondona M. SLURM: Simple Linux Utility for Resource Management. Job Scheduling Strategies for Parallel Processing. 2003:44–60. doi: 10.1007/10968987_3

[19] Nowogrodzki A. How to support open-source software and stay sane. Nature. 2019;571:133–4. doi: 10.1038/d41586-019-02046-0 31263262

[20] National Research Council (U.S.). Committee on the Science of Team Science, National Research Council (U.S.). Division of Behavioral and Social Sciences and Education. Enhancing the Effectiveness of Team Science. National Academies Press; 2015. 26247083

[21] Yanai I, Lercher M. Renaissance minds in 21st century science. Genome Biol. 2020;21:67. doi: 10.1186/s13059-020-01985-6 32169112 PMC7069164

[22] Cook CE, Stroe O, Cochrane G, Birney E, Apweiler R. The European Bioinformatics Institute in 2020: building a global infrastructure of interconnected data resources for the life sciences. Nucleic Acids Res. 2019;48:D17–23. 10.1093/nar/gkz1033 PMC6943058 31701143

[23] Cimini BA, Nørrelykke SF, Louveaux M, Sladoje N, Paul-Gilloteaux P, Colombelli J, et al. The NEUBIAS Gateway: a hub for bioimage analysis methods and materials. F1000Res. 2020;9:613. doi: 10.12688/f1000research.24759.1 32595963 PMC7308948

[24] Wilson G. Software Carpentry: Getting Scientists to Write Better Code by Making Them More Productive. Comput Sci Eng. 2006:66–9. doi: 10.1109/mcse.2006.122

[25] Goodwin SS. iBiology: communicating the process of science. Mol Biol Cell. 2014;25:2217–9. doi: 10.1091/mbc.E14-02-0756 25080124 PMC4116296

[26] Vines TH, Albert AYK, Andrew RL, Débarre F, Bock DG, Franklin MT, et al. The availability of research data declines rapidly with article age. Curr Biol. 2014;24:94–7. doi: 10.1016/j.cub.2013.11.014 24361065

[27] Wilkinson MD, Dumontier M, Aalbersberg IJJ, Appleton G, Axton M, Baak A, et al. The FAIR Guiding Principles for scientific data management and stewardship. Sci Data. 2016;3:160018. doi: 10.1038/sdata.2016.18 26978244 PMC4792175

[28] Wilson SL, Way GP, Bittremieux W, Armache J-P, Haendel MA, Hoffman MM. Sharing biological data: why, when, and how. FEBS Lett. Forthcoming [2021]. 10.1002/1873-3468.14067 PMC10390076 33843054

[29] Park Y, Greene CS. A parasite’s perspective on data sharing. Gigascience. 2018;7. doi: 10.1093/gigascience/giy129 30395209 PMC6258825

[30] Greene CS, Garmire LX, Gilbert JA, Ritchie MD, Hunter LE. Celebrating parasites. Nat Genet. 2017:483–4. doi: 10.1038/ng.3830 28358134 PMC5710834

[31] Byrd JB, Greene AC, Prasad DV, Jiang X, Greene CS. Responsible, practical genomic data sharing that accelerates research. Nat Rev Genet. 2020;21:615–29. doi: 10.1038/s41576-020-0257-5 32694666 PMC7974070

[32] Lau-Min KS, Asher SB, Chen J, Domchek SM, Feldman M, Joffe S, et al. Real-world integration of genomic data into the electronic health record: the PennChart Genomics Initiative. Genet Med. 2021;23:603–5. doi: 10.1038/s41436-020-01056-y 33299147 PMC8026392

[33] Eddy SR. “Antedisciplinary” science. PLoS Comput Biol. 2005;1:e6. doi: 10.1371/journal.pcbi.0010006 16103907 PMC1183512

